# Survival Trends and Long-Term Toxicity in Pediatric Patients with Osteosarcoma

**DOI:** 10.1155/2012/636405

**Published:** 2012-11-25

**Authors:** Melanie M. Hagleitner, Eveline S. J. M. de Bont, D. Maroeska W. M. te Loo

**Affiliations:** ^1^Department of Pediatric Hematology and Oncology, Radboud University Nijmegen Medical Centre, P.O. Box 9101, 6500 HB Nijmegen, The Netherlands; ^2^Department of Pediatric Hematology and Oncology, University Medical Centre Groningen, University of Groningen, P.O. Box 30.001, 9700 RB Groningen, The Netherlands

## Abstract

*Background*. This study was conducted to investigate the clinical characteristics and treatment results of osteosarcoma in pediatric patients during the past 30 years. Trends in survival rates and long-term toxicity were analyzed. *Procedure*. 130 pediatric patients under the age of 20 years with primary localized or metastatic high-grade osteosarcoma were analyzed regarding demographic, treatment-related variables, long-term toxicity, and survival data. *Results*. Comparison of the different time periods of treatment showed that the 5-year OS improved from 58.6% for children diagnosed during 1979–1983 to 78.6% for those diagnosed during 2003–2008 (*P* = 0.13). Interestingly, the basic treatment agents including cisplatin, doxorubicin, and methotrexate remained the same. Treatment reduction due to acute toxicity was less frequent in patients treated in the last era (7.1% versus 24.1% in patients treated in 1979–1983; *P* = 0.04). Furthermore, late cardiac effects and secondary malignancies can become evident many years after treatment. *Conclusion*. We elucidate the prevalence of toxicity to therapy of patients with osteosarcoma over the past 30 years. The overall improvement in survival may in part be attributed to improved supportive care allowing regimens to be administered to best advantage with higher tolerance of chemotherapy and therefore less chemotherapy-related toxicity.

## 1. Introduction 

 Osteosarcoma comprises 5% of all pediatric malignancies and is the most common primary bone cancer in children and adolescents. It is a highly aggressive tumor that usually involves the metaphysis of long bones and metastasizes primarily to the lung. Before the 1970s, the prognosis for patients with high-grade osteosarcoma was poor, with long-term survival rates of less than 20% [1]. Advances in adjuvant and neoadjuvant chemotherapy have improved the 5-year disease-free survival to more than 60% [2]. Since then different attempts have been made to further improve prognosis. However, it has been demonstrated that neither dose intensification nor addition of newer agents does improve survival [3–6]. Furthermore, patients that survive experience prolonged periods of rehabilitation after long periods of chemotherapeutic treatment and after often disabling surgery. Although the long-term toxicities have not been assessed completely, cardiotoxicity already emerged as a significant price of cure for survivors of osteosarcoma patients [7]. Monitoring trends in survival and the long term toxicities is essential to enhance current treatment regimens. 

 To gain more insight in the young patients, as osteosarcoma is predominantly being a disease that affects young patients, we investigate survival trends in this patient group and subsequently analyze the occurrence of long term toxicity over the last three decades. 

## 2. Material and Methods 

### 2.1. Patients

In total 130 pediatric patients were consecutively treated between 1979 and 2008 in two different centers: Radboud University Nijmegen Medical Centre and University Medical Centre Groningen. All patients under the age of 20 years with newly diagnosed, primary localized, or metastatic high-grade osteosarcoma were evaluated. Complete clinical and pathologic records and appropriate data regarding followup were present. Informed consent was obtained for all patients. 

### 2.2. Treatment

Information from the patients' medical charts about cumulative dosage of treatment, treatment response (good responders defined as <10% vital cells after neoadjuvant therapy), reduction of chemotherapy (defined as >15% reduction of planned chemotherapy), and surgical approach was collected. The overall period was split into subperiods relating to the major changes in protocol (1979–1983, 1984–2002, and 2003–2008). In the first era from 1979–1983 a T10-based multidrug regimen consisting of six cycles of doxorubicin 30 mg/m^2^ daily for 2-3 consecutive days, six cycles of cisplatin 120 mg/m^2^ administered by repeated 6 hours infusions, 8–12 cycles of high-dose methotrexate 8–12 mg/m^2^ with appropriate folinic acid rescue, and 1-2 cycles of BCD was used. From 1984 to 2002 trials with control arm treatment of six cycles of doxorubicin 25 mg/m^2^ daily for 3 days in combination with cisplatin 100 mg/m^2^ as a continuous infusion were used. The comparison arm consisted of only four doxorubicin/cisplatin courses and additionally 12 courses of high-dose methotrexate 12 mg/m^2^ with appropriate folinic acid rescue. In the last era from 2003 to 2008 patients was treated with six cycles of doxorubicine 25 mg/m^2^ daily for 3 days, four cycles of cisplatin 120 mg/m^2^ as a continuous infusion, and 12 cycles of high-dose methotrexate 8–12 g/m^2^ with appropriate folinic acid rescue. 

### 2.3. Survival

Overall survival (OS) and disease-free survival (DFS) were estimated using the Kaplan-Meier method [8]. OS was defined as the interval between diagnosis and death from any cause. Patients alive at the date of last followup were censored at that time point. DFS was defined as the interval between diagnosis and disease progression/recurrence. Patients without disease recurrence at the date of last followup were censored at that date. Cox's proportional hazard regression analysis was used to determine significance of differences in survival curves. Confidence intervals were calculated at the 95% level. We evaluated gender, location, stage, treatment, treatment response, and toxicity-induced reduction of chemotherapy as prognostic factors. Associations between outcome and potential predictors were evaluated with the Fisher's exact test for categorical variables. Statistical analysis was performed using SSPS software (version 16.0, SSPS Inc, Chicago, IL, USA). 

### 2.4. Long-Term Toxicities

Long-term toxicities after chemotherapeutic treatment were evaluated regarding development of secondary malignancies, cardiotoxicity, and ototoxicity. The length of time at risk for the secondary malignancy neoplasm (SMN) was calculated from the date of diagnosis of primary osteosarcoma until the date of diagnosis of the second malignancy. The cumulative incidence rate of second malignancies was calculated based on the method of Gray [9]. For the analysis of cardiac toxicity resulting from doxorubicin therapy, we included surviving patients who underwent serial echocardiographic evaluations before, during, and at least one followup echocardiogram after the initial treatment with doxorubicin. An echocardiogram at the end of chemotherapy was recommended annually for 5 years. Data concerning the fractional shortening (FS) value were collected. Cardiac dysfunction was defined as a decrease in FS <28% or a reduction of more than 10% [10]. Hearing loss, a complication of cisplatin was defined according to Brock Criteria [11] (grade 0: <40 dB at all frequencies; grade1: ≥40 dB at 8000 Hz; grade 2: ≥40 dB at 4000 Hz; grade3: ≥40 dB at 2000 Hz; grade 4: ≥40 dB at 1000 Hz). For this analysis only survivors were included with a baseline audiogram and an audiogram at the end of treatment. 

## 3. Results and Discussion

### 3.1. Patients

The median age at diagnosis was 14.4 years (range from 4.5 to 19.9 years). There were 72 male patients (55.4%; median age: 14.8 years; range from 6.3 to 19.9 years) and 58 female patients (44.6%; median age: 13.9 years; range from 4.5 to 19.1 years). At diagnosis, 29 patients (22.3%) had metastases and in 9 patients (6.6%) the tumor was located in the axial skeleton. 

### 3.2. Survival

#### 3.2.1. Overall Survival

**Figure 1:**
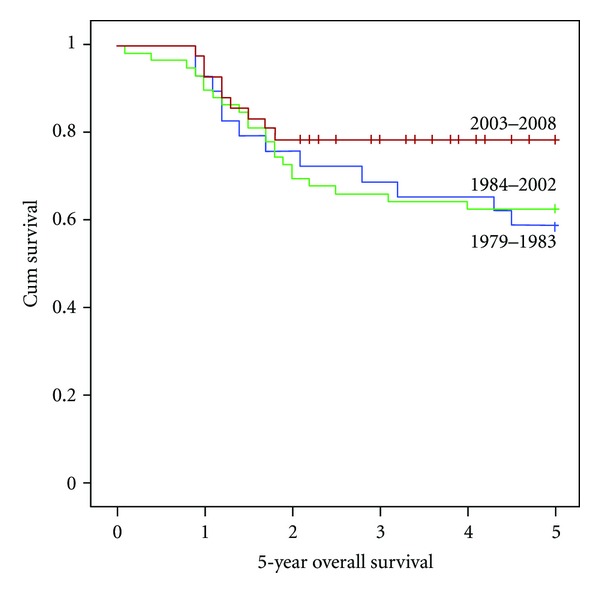
5-year overall survival in pediatric patients with osteosarcoma over three decades.

At a followup ranging between 4 and 33 years (median 8.9 years), 51 patients (39%) died. Of all deaths, 45 were directly related to osteosarcoma with 16 deaths due to progressive disease, eight due to local recurrence, and 21 after the development of distant metastases. The development of recurrent disease was predictive of death. Incidence of recurrences was relatively stable after 5 years with only three patients developing disease recurrence more than 5 years after diagnosis. Of the remaining six patients that died, four patients developed a second malignancy which was fatal, and two patients passed away due to anthracycline-induced cardiomyopathy. The 5-year OS of the whole population was 66.9% ± 0.15 the 10-year OS was 64.6% ± 0.23; the 20-year OS was 62.3% ± 0.82. These findings are in concordance with earlier reports in the literature [12, 13]. In total, 60 patients had a followup longer than 5 years, 38 patients were followed for more than 10 years, and a group of 20 patients was followed beyond 20 years. Comparison of the different time periods of treatment showed that the 5-year OS improved from 58.6% for children diagnosed during 1979–1983 to 78.6% for those diagnosed during 2003–2008 (*P* = 0.13; Figure 1). The distribution of known prognostic factors, such as metastatic disease at diagnosis, tumor location and good histologic response to neoadjuvant chemotherapy, was comparable regarding the different time periods (Table 1). 

#### 3.2.2. Disease-Free Survival

The 5-year DFS of the whole group was 59.2% ± 0.18. Patients with nonmetastatic osteosarcoma disease showed a DFS of 65.3% versus 37.9% in patients with metastases at diagnosis. During the past 3 decades the DFS of patients with metastases increased from 28.6% in 1979–1983, 40.0% in 1984–2002 to 42.9% in 2003–2008. The same trend is seen in patients with bad histologic response to neoadjuvant chemotherapy: 5-year DFS increased from 30.0% in 1979–1983, 57.6% in 1984–2002 to 65.4% in 2003–2008. Comparison of DFS in patients with axial involvement was not possible due to a small group. 

### 3.3. Treatment 

#### 3.3.1. Chemotherapeutic Treatment

In the first era, surgical resection was performed after 4 weeks of neoadjuvant chemotherapy, after 1984 resection was performed in the 10th week of induction therapy. Good histologic response to neoadjuvant chemotherapy was observed in 13.3% of patients treated with cisplatin and doxorubicin and in 29.4% of patients additionally treated with methotrexate (*P* = 0.083). Although an increased rate of good histologic responders was seen in the group additionally treated with methotrexate, comparing the regimen arms with and without methotrexate, no evident difference was seen in 5-year OS (HR = 0.75; 95% CI = 0.39–1.27; *P* = 0.24) or 5-year DFS (HR = 0.75; 95% CI = 0.43–1.29; *P* = 0.29). The effect of high-dose methotrexate in the treatment of osteosarcoma is not unambiguously proven in the literature. Several studies have shown a relationship between peak serum concentrations of methotrexate and improved histologic response [14–16]. However, a Cochrane systematic review comparing the effectiveness of methotrexate was unable to make clear conclusions due to the lack of randomized controlled trials using high-dose methotrexate as the only difference between the intervention and control group [17].

#### 3.3.2. Toxicity-Related Treatment Reduction

Chemotherapy was reduced due to severe bone marrow depression (*N* = 6), renal toxicity (*N* = 3), ototoxicity (*N* = 3), cardiotoxicity (*N* = 3), and adverse effects to methotrexate, like methotrexate encephalopathy (*N* = 1) or allergic reactions to methotrexate (*N* = 3). Interestingly, treatment reduction due to acute toxicity is less frequent in patients treated between 2003 and 2008 than in patients treated between 1984 and 1998 and in patients treated between 1979 and 1983 (*P* = 0.04, Table 1). 

#### 3.3.3. Surgical Treatment

 Analysis of surgical treatment method showed a rise from 26.9% of patients with limb salvage treatment in 1979–1983 to 69% in 2003–2008. The 5-year DFS rates were 57.8% for limb salvage treatment and 66.1% for amputation (HR = 1.35; 95% CI = 0.76–2.39; *P* = 0.30). The advancement of patients with limb salvage treatment could be expected considering that amputation patients might have had more advanced disease. A slightly but not significant improvement of DFS was seen in both surgical methods over the past three decades (data not shown).

### 3.4. Long-Term Toxicity

#### 3.4.1. Secondary Malignancy Neoplasm

In total, seven patients (5.3%) developed a second malignancy, with a median latency from original diagnosis to second malignancy of 12 years (range 2.4–23 years). Second malignancies were two patients with acute lymphatic leukemia, four patients with breast cancer, and one patient with gastric adenocarcinoma. Of the seven patients who developed secondary malignancy neoplasm (SMN), two patients had a history of Li-Fraumeni syndrome. Both patients developed SMN within 5 years after treatment of osteosarcoma. The 5- and 10-year estimated cumulative SMN incidence rates were 1.5% ± 1.1% and 3.1% ± 1.3%, respectively, which is similar to earlier studies in survivors of osteosarcoma [18, 19]. Overall, the incidence ratio of secondary malignancies in long-term survivors of osteosarcoma is much less compared to other malignancies, for example, Hodgkin disease [20]. However, the cohort has been followed for a median of 8.9 years, and it is likely that with increasing followup, second malignancies will emerge, due to a latency of 10 to 15 years. 

#### 3.4.2. Cardiotoxicity

In 46 patients sufficient data concerning baseline and followup echocardiogram was available. No patient had clinically evident cardiac disease before chemotherapy. Before starting therapy with doxorubicin the median FS value was 36%. In 8 patients the FS decreased <28% during therapy, with a median change in the percent FS value of 4% which is in concordance with earlier studies on this subject [21]. The long-term implications of doxorubicin-associated echocardiographic abnormalities are not fully understood, but at least one study suggests that echocardiographic abnormalities are progressive over time [22]. In our cohort, three patients with decreased FS during therapy remained to have cardiac dysfunction after finishing primary treatment. In total, 11 out of 46 patients developed cardiac dysfunction in the followup period. The median frequency of cardiac evaluation in the first five years was three (range: 1–5). The median latency of cardiac effects after finishing primary therapy was 219 days (range: from 30 days to 28.2 years). Six out of 11 patients with cardiac dysfunction experienced symptomatic heart failures, four of them more than 5 years after primary treatment and regular controls. Two patients died of cardiomyopathy and three of metastatic disease, which occurred after the diagnosis of cardiomyopathy. Affected patients were predominantly treated in the first two decades of our study realizing that the last cohort treated might not had enough followup to adequately evaluate this late sequelae. All patients received a mean cumulative doxorubicin dose of 450 mg/m^2^. 

#### 3.4.3. Ototoxicity

Cisplatin-associated hearing loss develops in the period when cisplatin is administered, and it has already been observed in patients treated with a single dose of 50 mg/m^2^ [23]. However, it is unlikely to improve over time, and as even mild hearing loss can have considerable impact on the social-emotional development of a child [24], it is considered to be one of the late effects in the treatment of osteosarcoma patients. Of the 79 survivors in our study, 62 (78.5%) had an audiogram at the end of treatment. Preexisting hearing loss was not reported in one of these patients. Of the 62 survivors with an audiogram, 31 patients (50%) had no hearing loss, 23 patients (37.1%) were Brock grade I, and 7 patients (11.1%) developed severe hearing loss (Brock grades 3 and 4). All patients with severe hearing loss required hearing aids. No difference was seen in cumulative dosage of cisplatin comparing patients with severe hearing loss (cumulative dosage: 407 mg/m^2^) versus patients with grade 0,1 or 2 hearing loss (cumulative dosage: 442 mg/m^2^,  *P* = 0.55). Furthermore, no difference in prevalence of severe ototoxicity was seen between the drug regimens using either 600 mg/m^2^ or 480 mg/m^2^ cisplatin (*P* = 0.25) or continuous infusion versus repeated 6-hour infusions (*P* = 0.76).

Like other studies in childhood cancer [25] we showed an improved survival of patients with osteosarcoma over the past decades. This improvement is seen in all subgroups irrespective of known prognostic factors like metastases at diagnosis or histological response to neoadjuvant therapy. This increase in childhood cancer survival is often attributed to more effective treatment strategies. However, in our study the treatment agents and dosage basically remained the same over the past 30 years with cumulative doses of doxorubicin 450 mg/m^2^, cisplatin 480 or 600 mg/m^2^, and high-dose methotrexate 96–144 g/m^2^ appropriate to age. Furthermore, there was no difference in method of administration regarding doxorubicin and high-dose methotrexate. Cisplatin was administered as a 6-hour infusion during 1979–1983 in our study and then as continuous infusion. While continuous cisplatin infusions are observed to be considerably ototoxic (Lanvers-Kaminsky PBC 2006 [26]), we found no difference in our study. Therefore, other factors like empiric use of broad spectrum antibiotics in case of neutropenic fever, pain management, antiemetics, nutrition, blood banking, and improved pediatric intensive care might have improved the acceptability of the current treatment. On the other hand, with an increasing number of cancer survivors detection of late effects of osteosarcoma therapy is required. In this study, we show that late cardiac effects and SMN can become evident many years after treatment and can have profound impact on cancer survivors. 

 In conclusion, these data elucidate the prevalence of toxicity to therapy in children with osteosarcoma. Further studies concerning the management of long-term toxicities are needed to finally improve survival rates in patients with osteosarcoma. 

## Figures and Tables

**Table 1:** Distribution of prognostic factors over three decades.

Prognostic factor (*N*)	1979–1983	1984–2002	2003–2008	*P*
	*N* = 29	*N* = 59	*N* = 42	
Metastases at diagnosis	24.1% (7)	25.4% (15)	16.7% (7)	0.58
Tumor in axial skeleton	6.9% (2)	8.5% (5)	4.8% (2)	0.51
Good histologic response	24.1% (7)	23.7% (14)	35.7% (15)	0.55
>15% reduction due to toxicity	24.1% (7)	20.3% (12)	7.1% (3)	0.04
